# DNA Methylation Dynamics in Plant Abiotic Stress Response: Mechanisms, Memory, and Breeding Applications

**DOI:** 10.3390/genes17030301

**Published:** 2026-02-28

**Authors:** Huanqing Huang, Chenyu Guo, Shiping Cheng, Zhe Wang

**Affiliations:** 1 School of Geography and Environment Engineering, Pingdingshan University, Pingdingshan 467000, China; 2865@pdsu.edu.cn; 2 Henan Province Key Laboratory of Germplasm Innovation and Utilization of Eco-Economic Woody Plant, School of Chemistry and Environmental Engineering, Pingdingshan University, Pingdingshan 467000, China; guo_7368@163.com (C.G.); shipingcheng@163.com (S.C.); 3 Postdoctoral Station of Crop Science, Henan Agricultural University, Zhengzhou 450046, China

**Keywords:** DNA methylation, abiotic stress, epigenetic memory, RdDM, crop breeding

## Abstract

Abiotic stresses such as drought, salinity, extreme temperatures, and heavy metal contamination severely limit global crop productivity and threaten food security. Plants have evolved epigenetic strategies, particularly DNA methylation, to perceive, adapt to, and memorize environmental challenges. This review systematically elucidates the dynamic regulatory mechanisms of DNA methylation—including establishment via RNA-directed DNA methylation (RdDM), maintenance by methyltransferases (MET1, CMT), and active removal by demethylases (*ROS1*)—in plant responses to diverse abiotic stresses. We highlight how stress-induced methylation reprogramming modulates gene expression, chromatin states, and physiological adaptations, contributing to both somatic and transgenerational stress memory. Furthermore, we discuss advanced detection technologies for profiling methylation patterns and evaluate their applications in epigenetic breeding, such as exploiting heritable epialleles, RdDM-based gene silencing, and methylation markers for heterosis prediction. Despite significant progress, translating epigenetic insights into predictable breeding tools remains challenging. Future efforts should focus on establishing causal links between methylation changes and stress phenotypes, improving epigenome editing precision, and integrating multi-omics approaches for the development of climate-resilient crops. This work provides a comprehensive epigenetic perspective for enhancing crop adaptability and sustainable agriculture.

## 1. Introduction

**Figure 1:**
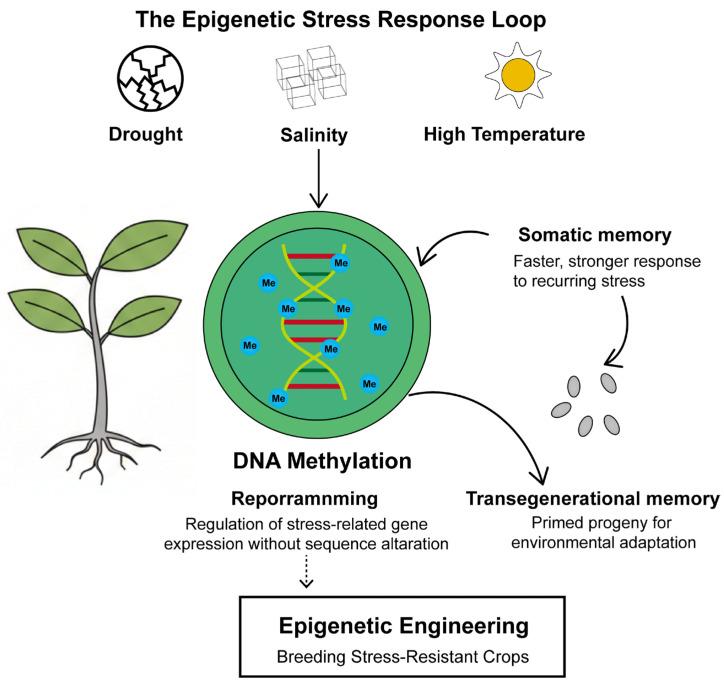
Epigenetic regulation of plant responses to abiotic stress.

Plants are constantly challenged by abiotic stresses such as drought, salinity, and extreme temperatures, which are major drivers of global crop yield losses and threaten food security [1,2,3,4]. To cope with these environmental fluctuations, plants have evolved sophisticated adaptive strategies, among which epigenetic regulation—particularly DNA methylation—has emerged as a central mechanism [5,6]. As a stable yet dynamic epigenetic mark, DNA methylation regulates gene expression without altering the DNA sequence, enabling rapid and reversible responses to stress [7]. For example, drought memory genes in *Arabidopsis thaliana* remain poised during recovery [8]. Transgenerational memory transmits stress information to offspring, enhancing their adaptability [9]. Various abiotic stresses have been shown to induce genome-wide reprogramming of DNA methylation patterns, thereby modulating the expression of stress-responsive genes and contributing to plant tolerance [10,11,12]. Therefore, systematically elucidating the mechanisms of DNA methylation in plant abiotic stress responses not only deepens our understanding of plant environmental adaptation from an epigenetic perspective but also provides a theoretical foundation for developing stress-resistant crop varieties through epigenetic engineering and breeding strategies (Figure 1).

This figure outlines the central role of DNA methylation in plant abiotic stress responses, encompassing stress signal perception, dynamic methylation changes (establishment, maintenance, removal), gene expression reprogramming, stress memory formation, and breeding applications.

## 2. The Dynamic Regulatory System of Plant DNA Methylation

### 2.1. Establishment, Maintenance, and Removal of Plant DNA Methylation

Plant DNA methylation is a highly complex and dynamically regulated epigenetic process, exhibiting both conservation and specificity across different plant lineages [13,14]. Its establishment primarily relies on the RNA-directed DNA methylation pathway, which utilizes plant-specific RNA Polymerase IV to produce 24-nt siRNAs and Pol V to generate scaffold lncRNAs, thereby recruiting the *AGO4*-*DRM2* complex for de novo methylation [15,16]. In cereal crops, the RdDM pathway shows species-specific variations. For instance, in rice, besides the canonical pathway, a non-canonical pathway exists where Pol II transcripts are processed by DCL3 into 24-nt miRNAs to guide methylation [17]. In maize, the loss of Pol IV leads to the upregulation of genes with nearby transposable elements, highlighting RdDM’s core role in maintaining chromatin boundaries. Meanwhile, a Pol II–RDR6-dependent pathway can partially compensate for TE silencing upon Pol IV loss, and the RdDM pathway is closely linked to subgenome dominance [18] DNA methylation maintenance differs by sequence context. At CG sites, it is primarily maintained by *MET1* homologs post-replication [19]. At non-CG sites, a reinforcing loop exists between DNA methylation and histone H3K9 methylation in *Arabidopsis*: *CMT3* and *CMT2* mediate CHG and CHH maintenance in long TEs, respectively, while RdDM mainly maintains CHH at short TEs and TE edges [20]. This feedback mechanism is conserved but specialized in cereals. For example, maize *MET2* prefers linker DNA [21,22], and the chromatin remodeler *DDM1* facilitates maintenance by promoting DNA accessibility in heterochromatin [23,24].

DNA demethylation is primarily mediated by *ROS1* family DNA glycosylases, which excise 5mC and initiate base excision repair to prevent aberrant methylation spread [25,26]. In rice, *OsROS1* also participates in erasing methylation at specific gene regions. This process coordinates with histone acetylation and chromatin remodeling. In summary, the core machinery of plant DNA methylation is conserved but exhibits species-specific adaptations, which likely contribute to differential environmental adaptation.

### 2.2. Regulatory Logic: Signal Input, Dynamic Properties, and the Reader–Writer Network

#### 2.2.1. Molecular Basis of Dynamicity and Memory

The “dynamicity” and “memorability” of plant DNA methylation stem from the interplay of three key processes: de novo establishment writes new information, post-replicative maintenance ensures inheritance during cell division, and active removal allows erasure and correction [27]. The symmetry of CG and CHG sequences enables the precise restoration of their methylation states using the parental strand as a template, enabling short-term somatic memory. More profoundly, in heterochromatin, a self-reinforcing positive feedback loop exists between DNA methylation and histone H3K9 methylation: DNA methylation is recognized by *SUVH* family “readers” that guide new H3K9me deposition [28,29], while H3K9me is specifically recognized by the chromodomains of methyltransferases like *CMT2/3*, recruiting them to maintain or reinforce DNA methylation [21]. The10refore, this self-reinforcing cycle not only stabilizes gene silencing but also establishes a form of long-term epigenetic memory that can be transmitted across cell generations.

#### 2.2.2. Upstream Signal Input and Targeting

The plant DNA methylation system achieves spatiotemporal specificity and self-maintenance through the precise decoding of upstream molecular signals, with core inputs originating from endogenous chromatin environments and system products. As crucial developmental signals, specific histone modification patterns directly regulate the activation of RNA-directed DNA methylation pathways. For instance, the *SHH1* protein functions as an integration factor whose SAWADEE domain simultaneously recognizes histone H3 lysine 9 methylation and lysine 4 non-methylation states, thereby guiding RNA polymerase IV complexes to heterochromatic regions to establish site-specific de novo methylation [30,31]. Concurrent studies show that proteins such as ZMP perform analogous regulatory functions in specific genomic regions by recognizing H3K4me0 modifications, further enriching the chromatin state-based targeting network [32].

Notably, DNA methylation modifications themselves constitute self-reinforcing signaling circuits. *SUVH2* and *SUVH9*, as methylated cytosine recognition proteins, specifically bind methylated DNA via their SRA domains, subsequently recruiting the DDR chromatin remodeling complex composed of *DRD1*, *DMS3*, and *RDM1* to target RNA polymerase V for transposon silencing [33]. This “silencing begets deeper silencing” positive feedback mechanism enhances heterochromatin stability and environmental resilience. Research demonstrates that DNA methylation dynamically responds to various environmental stresses. Under biotic stress, pathogen infection induces hypomethylation in specific host genomic regions, regulating defense gene expression [34,35,36]. Abiotic stresses similarly trigger methylome reprogramming: salt stress alters methylation states of *LCR* and *TPS4* genes in Brassica napus [37]; drought induces hypomethylation in the *Asr2* gene promoter in tomato [38]; cold stress modifies CHH-context methylation patterns in *Arabidopsis* [39,40,41]; heavy metal stress regulates DNA methylation states in ferns and algae [42,43]; and light dynamics optimize photosynthetic function through methylation remodeling in transposable element regions [44]. Stress signals drive specific epigenome reprogramming by modulating the activities of methyltransferases (*MET1*, *DRM2*) and demethylases (*ROS1*, *DME*) [45]. This dynamic regulatory mechanism enables plants to develop highly specific epigenetic response strategies tailored to distinct stress characteristics. For example, methylation remodeling induced by drought versus salt stress exhibits significant differences in target gene selection, physiological pathway regulation, and hormonal network interactions, reflecting the precision and plasticity of plant environmental adaptation.

#### 2.2.3. From Epigenetic Modification to Functional Output: The Regulatory Loop

Through upstream signal integration and the operation of the internal “reader–writer” network, changes in DNA methylation are ultimately translated into stable biological outputs. Whether through newly established methylation via RdDM [46,47,48,49] or structural changes triggered by methylation “readers” (e.g., *SUVH2/9*) recruiting chromatin remodeling complexes (e.g., *DDR*) [50,51,52], the common outcome is the shaping of the local chromatin environment. High levels of DNA methylation, particularly in coordination with repressive histone marks such as H3K9me2, lead to chromatin condensation into transcriptionally inaccessible heterochromatin. This state stably suppresses transposable element activity, safeguarding genome integrity [53,54]. When such modifications occur in promoter or enhancer regions, they impede transcription factor binding and result in the downregulation of associated genes [55].

Conversely, active demethylation mediated by *ROS1* removes repressive marks and drives chromatin toward an active state. *ROS1* functions as a bifunctional DNA glycosylase/lyase that directly excises 5mC and restores unmethylated status through base excision repair, a process potentially coordinated with downstream repair enzymes (e.g., *APE1L*, *ZDP*) to promote local chromatin opening [56,57,58]. The loci of core transcriptional activators often maintain an open chromatin state, providing space for regulator binding—a basis for rapid and tunable gene expression [59].

**Figure 2:**
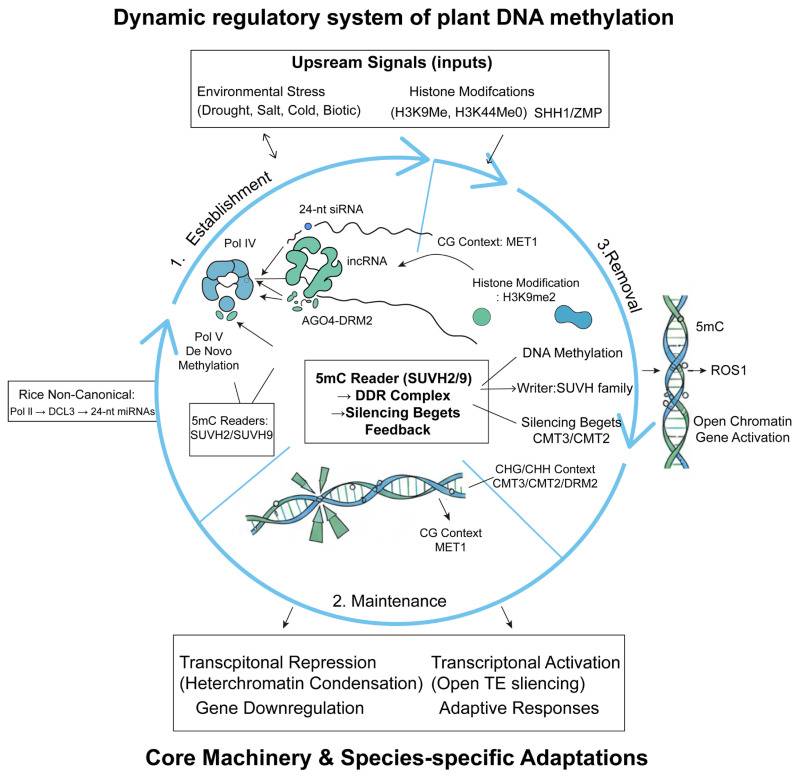
The dynamic regulatory system of plant DNA methylation: molecular mechanisms of establishment, maintenance, and removal, and their regulatory networks.

Collectively, the studies reviewed here demonstrate that the dynamic DNA methylation system—comprising establishment (RdDM), maintenance (*MET1/CMT*), and removal (*ROS1*)—provides a versatile molecular basis for plants to perceive, integrate, and memorize environmental signals. This epigenetic programmability allows for stress-specific reprogramming of the methylome, which in turn drives adaptive transcriptional responses. Therefore, harnessing this system—through targeting key regulators like *DRM2*, *CMTs*, or *ROS1*—represents a promising epigenetic engineering strategy for developing crop varieties with enhanced resilience to diverse abiotic stresses. Future research should focus on decoding how specific stress signals (e.g., drought, salinity, temperature) are transduced into precise methylation remodeling instructions to enable predictive and precise manipulation for breeding (Figure 2).

This figure illustrates the core regulatory mechanisms of plant DNA methylation: RdDM-mediated establishment, MET1/CMT-catalyzed maintenance, and ROS1-mediated active demethylation, along with the regulatory logic of upstream signal input and chromatin state output.

## 3. The Role of DNA Methylation in Various Abiotic Stresses

### 3.1. Drought Stress

DNA methylation serves as a core epigenetic mechanism in plant responses to drought stress. Drought signals trigger epigenomic reprogramming, establishing new methylation marks at specific gene loci via the RdDM pathway [60,61,62]. Subsequently, methylation in different sequence contexts is maintained by specialized enzymatic systems: *MET1* maintains CG methylation, *CMT3* collaborates with *SUVH4* to sustain CHG methylation, while *CMT2* and ongoing RdDM activity are responsible for CHH site maintenance [63]. Similar mechanisms are observed in other species: decreased global 5-mC content in *Arabidopsis* is linked to the *DCL2/DCL3* pathway [64]; reduced genome-wide methylation in Populus tomentosa correlates with an altered expression of genes like *GATA9*, promoting ABA and osmolyte accumulation [65]; and differential methylation in the *ZmEXP2* gene promoter influences root architecture in maize [66]. These changes coordinately regulate multiple physiological processes including ABA signaling, ROS scavenging, osmotic balance, and root development, enhancing plant drought tolerance. Importantly, some drought-induced methylation variants can be stably inherited, forming transgenerational stress memory that confers stronger adaptive capacity in progeny [67].

### 3.2. Salt Stress

Salt stress induces tissue-specific methylation changes in rice, with significant hypomethylation in roots and more subtle alterations in leaves [68]. This response exhibits cultivar specificity: salt-tolerant genotypes (e.g., Nonabokra) often display hypermethylation, whereas sensitive ones (e.g., *IR64*) show demethylation [69]. Molecularly, RdDM pathway components such as *RDM16* modulate DNA methylation by affecting Pol V transcript levels, thereby enhancing salt tolerance [70]. Methylation status directly influences the expression of salt tolerance-related genes; for instance, the *OsBZ8* locus is hypomethylated and highly expressed in the tolerant Nonabokra cultivar [71]. These methylation variations likely help maintain ion homeostasis by regulating the expression of Na^+^ transporters (e.g., *OsHKT1;5*) and ABA signaling pathway genes, offering potential targets for breeding salt-tolerant varieties.

### 3.3. Temperature Stress (Cold/Heat)

Under both high- and low-temperature stress, DNA methylation, as a dynamic and reversible epigenetic modification, plays a crucial role in plant stress response and memory. This system responds to temperature signals, achieving stress-specific methylation remodeling, which in turn regulates gene expression and crop adaptability. Cold stress widely induces DNA methylation changes involved in cold acclimation. In Populus simonii and Chorispora bungeana, cold stress-induced DNA methylation alterations at specific gene loci are directly related to enhanced freezing tolerance [72,73]. In *Arabidopsis*, DNA methylation cooperates with histone modifications to regulate cold responses. For instance, the histone deacetylase *HDA6* plays a key role in cold acclimation by modifying chromatin states [74]. Genome-wide studies confirm that cold-induced changes in chromatin accessibility are closely associated with dynamic alterations of histone marks (e.g., H3K4me3 and H3K27me3) at cold-responsive genes [75]. In rice, cold stress similarly triggers dynamic methylation responses. Altered methylation status in the *OST1* gene promoter of cold-tolerant cultivars mediates the ICE-CBF-COR cold signaling pathway, activating cold-resistant gene expression. Genome-wide analysis also identified 51 genes whose methylation and expression levels change synchronously in cold-tolerant varieties [76].

Heat stress also activates dynamic methylation responses. In *Arabidopsis*, heat can trigger active demethylation at regulatory regions of heat shock genes (e.g., *HSPs*), promoting their expression [77]. Rice studies show that heat stress alters the methylation status of genes related to heat shock proteins and antioxidant enzymes, affecting their transcriptional plasticity [78]. Significant DNA methylation differences exist between heat-tolerant and heat-sensitive wheat genotypes, directly correlating with thermotolerance [79]. The RdDM pathway is essential for maintaining basal thermotolerance [80]. Furthermore, heat stress activates retrotransposons (e.g., *ONSEN* in *Arabidopsis*), whose transcription is regulated by the methylation status of *HSEs* in their promoters. This activation can be maintained in progeny of siRNA biogenesis-deficient mutants, demonstrating the transgenerational potential of heat stress epigenetic memory [81,82]. Regarding rice responses to high temperature, research shows that the expression of the heat-sensitive gene *OsFIE1* is temperature-regulated, involving changes in both DNA methylation and histone H3K9me2 modifications. Heat also causes the disruption of CHH methylation patterns in anthers, and inhibition of DNA methylation can lead to pollen sterility in heat-sensitive varieties [83]. These findings indicate that temperature stress regulates crop stress tolerance by altering the methylation status of key genes. The DNA methylation system can decode different temperature signals to achieve specific epigenetic remodeling. This dynamic and heritable mechanism serves as a core molecular foundation for plants to build stress memory and rapidly adapt to environmental changes.

### 3.4. Carbon Stress

The rising atmospheric CO_2_ concentration has established elevated CO_2_ as a significant abiotic stress factor affecting plant growth and development [84]. Similar to traditional stresses like drought and heat, high CO_2_ can trigger not only immediate physiological responses but also potentially induce transgenerationally heritable “stress memory” [85,86]. In plants, DNA methylation serves as a core molecular carrier of this epigenetic memory, playing a pivotal role in responding to the stress signal and in the establishment and maintenance of memory marks [87,88]. Recent research reveals that plant transgenerational epigenetic adaptation to high CO_2_ follows a precise two-step cooperative regulatory model. Firstly, the establishment of high CO_2_-induced DNA methylation marks is primarily driven by the RdDM pathway. Studies in *Arabidopsis* show that exposure to high CO_2_ induces widespread hypermethylation across the genome simultaneously in CG, CHG, and CHH sequence contexts, a pattern characteristic of the RdDM pathway. Genetic experiments confirm that this DNA hypermethylation response is completely abolished in mutants lacking core RdDM components (e.g., pol IV mutants). This directly proves that RdDM acts as the critical bridge linking the high CO_2_ stress signal to the establishment of DNA methylation marks [89]. Secondly, the transgenerational inheritance of newly established methylation marks by RdDM requires the function of maintenance methyltransferases *CMT2* and *CMT3*. Observations in *Arabidopsis CMT2*/*CMT3* double mutants show that, although stressed parental plants can still generate a partial (albeit weakened) increase in methylation via RdDM, these newly established marks fail to transmit to the progeny. Concurrently, the progeny lose the transgenerational accelerated growth phenotype. This result clearly indicates that *CMT2* and *CMT3*, as key “maintainers,” are essential for converting the initial “written” stress memory by RdDM into a heritable long-term memory [90]. It is worth noting that epigenetic regulation triggered by exogenous carbon nanomaterials (e.g., Carbon Dots, CDs), also related to “carbon,” follows a similar molecular logic. In rice, CDs treatment similarly induces genome-wide DNA hypermethylation (particularly in CHG/CHH contexts), accompanied by the upregulated expression of maintenance methyltransferase genes like *OsCMT1* and *OsCMT2* [91]. This further corroborates the central executor role of CMT family enzymes in integrating carbon-related environmental signals and remodeling the DNA methylation landscape.

### 3.5. Nutrient Stress

#### 3.5.1. Nitrogen Stress

Nitrogen stress affects plant growth via epigenetic mechanisms. In *Arabidopsis*, low-nitrogen treatment influences methylation at eight SNP sites on chromosome 1 related to shoot growth, affecting the identified gene regions. The participation of key RdDM components like *RDR2* is associated with biomass accumulation under low nitrogen [92]. In rice, low nitrogen upregulates methyltransferase genes (e.g., *MET1*, *DRM1*, *DRM2*), leading to altered genome-wide methylation patterns that are heritable and enhance low-nitrogen tolerance in offspring [93]. Chronic (one-generation) exposure to low-nitrogen stress in rice induces alterations in DNA methylation patterns, with approximately 50% of these changes being heritable to non-stressed progeny [89]. The process of transgenesis and tissue culture also induces DNA methylation changes in progeny rice, as observed in lines carrying *OsNAR2.1*-related constructs [94]. Reduced nitrogen content in parental seeds also causes DNA methylation changes in progeny rice [95]. Cytosine methylation changes around transposable elements under natural nitrogen stress are significantly higher than in other genomic regions, suggesting TEs are sensitive targets in nutrient stress response [96].

#### 3.5.2. Phosphorus Stress

Phosphorus starvation leads to genome-wide methylation alterations. In *Arabidopsis*, 20% and 86% of differentially methylated regions in shoots and roots, respectively, are hypermethylated, closely correlating with the expression patterns of phosphorus stress-responsive genes [97]. The deubiquitinase *OTU5* is crucial for establishing DNA methylation patterns under phosphorus stress [98]. In tomato, phosphorus stress increases overall methylation levels, with differentially methylated sites highly correlated with TE distribution [99]. In Populus trichocarpa, differential methylation associates with locus-dependent microRNAs [100]. In soybean, low-phosphorus-induced CHH methylation changes are particularly prominent in TE regions and are precisely guided by siRNAs [101]. In rice, phosphate starvation-induced methylation changes preferentially occur in TE regions; in the absence of *DCL3a*, these TEs become hypermethylated to suppress their transcription [102].

#### 3.5.3. Trace Element Stress

Maize experiences an extensive loss of DNA methylation, primarily in TE regions, alongside the suppressed activity of maintenance methyltransferases and a sharp decrease in small RNAs associated with DNA methylation [103]. Under iron deficiency, rice exhibits widespread hypermethylation, especially in CHH contexts. The transcriptional abundance of iron deficiency-induced genes positively correlates with 24nt siRNA abundance, suggesting siRNA-guided methylation may participate in iron homeostasis regulation [104]. In barley, iron deficiency causes differential methylation in 11 DNA bands, and the methylation/demethylation pattern upon iron resupply closely resembles that under continuous deficiency, indicating the potential heritability of this modification [105]. Using MSAP analysis, researchers found that silicon treatment alters both total methylation levels and the patterns of hemimethylation versus symmetric methylation in a zinc concentration-dependent manner. These findings suggest that silicon can reshape the plant’s epigenomic landscape, thereby influencing molecular stress responses and potentially contributing to the establishment of “epigenetic memory” for enhanced heavy metal tolerance [106]. A similar pattern emerged under salt stress, where barley plants also exhibited significant changes in DNA methylation levels following exogenous silicon application. MSAP analysis showed that foliar sprays of moderate (0.1%) and high (0.2%) silicon concentrations markedly reduced total methylation compared to plants treated with NaCl alone, accompanied by distinct shifts in hemimethylation and symmetric methylation. This epigenetic response aligned with improved physiological traits—such as higher chlorophyll content, gas exchange, and chlorophyll fluorescence—suggesting that silicon may enhance salt tolerance through DNA demethylation and the activation of stress-related genes, potentially laying the groundwork for “epigenetic memory” [107]. Iron stress can induce DNA methylation changes in plants, with this epigenetic regulation documented across multiple species. In rice, iron deficiency leads to accumulation of CHH methylation in both roots and shoots, with hypermethylation occurring at the promoter regions of key iron homeostasis regulators *OsIRO2* and *OsbHLH159*, thereby affecting their expression; treatment with methyltransferase inhibitors represses genes involved in the iron signaling pathway, and rice mutants with reduced CHH methylation levels exhibit growth inhibition under iron-deficient conditions, indicating that DNA methylation plays a crucial role in rice adaptation to iron stress [108]. In *Arabidopsis*, cadmium exposure induces DNA methylation changes in roots, and the inhibition of DNA demethylation enhances plant tolerance to cadmium toxicity, an effect associated with improved iron nutrition, revealing an additional link between iron homeostasis regulation and epigenetic modifications under heavy metal stress [104]. Furthermore, in barley, iron deficiency leads to a general reduction in CG methylation, and the methylation/demethylation pattern upon iron resupply closely resembles that under continuous deficiency, indicating the potential heritability of this modification [109]. In *Arabidopsis*, *SKB1*-mediated histone *H4R3* symmetric dimethylation negatively regulates the expression of Ib subgroup *bHLH* genes, thereby participating in the maintenance of iron homeostasis, further expanding our understanding of the mechanisms underlying epigenetic regulation of iron homeostasis [105].

### 3.6. Light Stress

Light stress, particularly dynamic fluctuations in light intensity, is an important environmental signal influencing plant DNA methylation. Variations in light can trigger epigenetic reprogramming by inducing reactive oxygen species (ROS) production, triggering chloroplast signals, and regulating small RNA expression. Tobacco mutants overproducing H_2_O_2_ exhibit genome-wide DNA hypomethylation [110], and loss of the chloroplast signal protein *MSH1* also causes genome-wide methylation reprogramming [111]. Small RNAs induced by high light can influence methylation patterns via the RdDM pathway [112]. Notably, in *Arabidopsis*, short-term repetitive excess light stress did not induce significant methylation changes, with only a small number of stress-related differentially methylated regions observed [113], indicating that short-term, minor light fluctuations have a limited overall impact on methylation. Different light qualities (e.g., white, blue, red light, and darkness) can induce specific changes in genomic DNA methylation. In tomato 7B-1 male-sterile mutants and their wild type, the methylation status of various functional genes changes differentially under different light qualities, and these alterations are more pronounced in mutants defective in light signaling pathways [114]. Methylation levels under darkness and red light are generally higher than under white and blue light, suggesting that light quality may finely tune gene expression through differential methylation mechanisms.

Recent research indicates that light fluctuation patterns are a primary driver of DNA methylation reprogramming. The induced differentially methylated regions are significantly enriched in transposable elements (especially retrotransposons) and regulate physiological adaptation by affecting the expression networks of neighboring genes. Functional validation shows that the loss of CG methylation specifically enhances photosynthetic efficiency under fluctuating light, and the key gene MCCA, regulated by light patterns, is involved in energy partitioning within the photosynthetic apparatus. This establishes a complete regulatory chain: “light dynamic signal–TE methylation remodeling–neighboring gene regulation–photosynthetic physiological adaptation.”

### 3.7. Ozone Stress

Recent research has significantly advanced our understanding of how elevated tropospheric ozone (O_3_) impacts plant systems, particularly through epigenetic modifications. As a potent phytotoxic pollutant, O_3_ induces oxidative stress by generating ROS upon entering plant tissues through stomata, leading to cellular damage, reduced photosynthetic efficiency, and ultimately compromised growth and yield [115,116]. For instance, studies on wheat cultivars have demonstrated that elevated O_3_ increases stomatal flux and the accumulation of superoxide radicals (O_2_^−^) and hydrogen peroxide (H_2_O_2_), resulting in significant lipid peroxidation [116]. Rice (*Oryza sativa* L.), a critical global food crop, demonstrates high sensitivity to O_3_ exposure, with studies in East Asia indicating potential yield losses exceeding 40% under polluted conditions, posing substantial risks to agricultural productivity and food security. In response to such environmental challenges, plants deploy complex adaptive strategies where epigenetic regulation, especially dynamic DNA methylation changes, plays a fundamental role in modulating gene expression and phenotypic plasticity without altering the primary DNA sequence [117,118]. Investigations into the epigenetic dimensions of O_3_ stress have revealed concentration-dependent effects on both physiological traits and molecular patterns in rice. Experimental studies utilizing open-top chamber systems to simulate different O_3_ levels (80, 160, and 200 nmol·mol^−1^) showed that lower concentrations (80 nmol·mol^−1^) could transiently promote certain agronomic parameters such as flag leaf length and panicle length, whereas higher concentrations (160 and 200 nmol·mol^−1^) consistently caused visible leaf injury, including chlorosis and necrotic lesions, alongside the significant suppression of growth metrics like hundred-grain weight [119]. At the molecular level, methylation-sensitive amplified polymorphism analyses conducted across these treatments detected rapid and widespread alterations in genomic DNA methylation, affecting approximately 15% of the assayed sites within days of exposure. Notably, the overall methylation landscape shifted towards reduced methylation levels (demethylation) over a 30-day exposure period, with these changes being more pronounced at higher O_3_ concentrations. Sequence context analysis further indicated that CNG sites (specifically CHG methylation) were predominant hotspots for these stress-induced methylation variations, corroborating earlier observations that such genomic contexts are particularly labile under abiotic stress conditions [120]. The functional implications of these epigenetic alterations were explored by isolating and sequencing differentially methylated DNA fragments. Among 153 cloned sequences showing methylation polymorphism, nine demonstrated high homology to known rice genes, eight of which were in a demethylated state under O_3_ stress. This bias towards demethylation suggests the potential for the transcriptional activation of associated genes. The annotated functions of these sequences point to a coordinated stress response network: genes like purple acid phosphatase and L-isoaspartate methyltransferase are involved in ROS detoxification and the repair of oxidative damage to proteins; phosphoenolpyruvate carboxylase plays roles in carbon metabolism and stomatal regulation; zinc finger proteins and KH-domain-containing proteins are often implicated in transcriptional regulation and RNA metabolism; and even transposon-related sequences showed altered methylation, hinting at possible genome-wide destabilization or adaptive mobilization under severe stress. This pattern indicates that O_3_ stress triggers not just random epigenetic noise but a directed, albeit complex, reprogramming likely aimed at activating defense pathways, enhancing metabolite synthesis for osmotic adjustment, and potentially repairing oxidative damage.

This body of work establishes DNA methylation as a dynamic and responsive layer of regulation in rice confronting O_3_ pollution. The observed demethylation, particularly at specific functional loci, may facilitate the expression of a suite of adaptive genes, thereby modulating the plant’s physiological response to oxidative stress. These findings significantly enrich the molecular narrative of plant-O_3_ interactions, moving beyond descriptive toxicology to reveal active epigenetic adaptation mechanisms. They provide a crucial conceptual framework and candidate gene targets for future research aimed at unraveling the precise signaling pathways linking O_3_ perception to epigenetic remodeling.

### 3.8. Heavy Metal Stress

Heavy metal stress (particularly cadmium, Cd) induces significant DNA methylation changes in rice. Cd stress leads to the detection of numerous differentially methylated regions (DMRs) genome-wide, with over 2320 non-redundant DMRs identified, and hypermethylated genes constituting a higher proportion [121]. These methylation changes are closely linked to the expression of heavy metal transporter genes. For example, the transcribed region of the metal detoxification transporter gene *OsZIP1* is demethylated, and its overexpression reduces Cd accumulation in rice [122]. Overexpression of the heavy metal-responsive protein *OsHMP* also involves altered DNA methylation status, enhancing rice growth under Cd stress [123]. Notably, some heavy metal stress-induced methylation variants can be inherited across generations, forming epigenetic memory. For instance, the methylation status of the heavy metal transporter-related retrotransposon *Tos17* remains stable over multiple generations [124]. This heritable methylation modification provides an epigenetic basis for long-term adaptation to heavy metal-contaminated environments.

**Figure 3:**
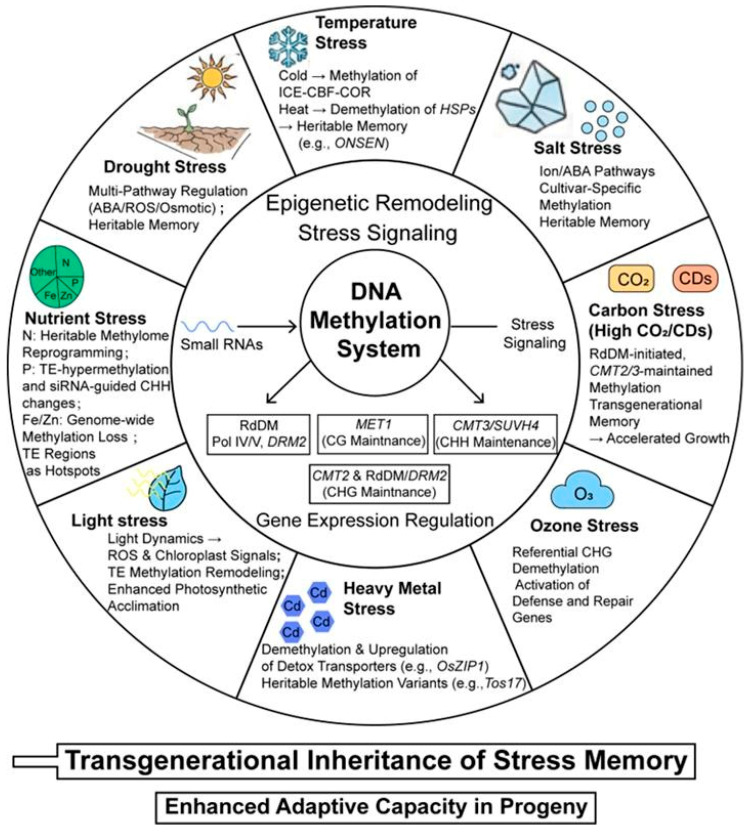
Landscape of stress-specific DNA methylation reprogramming and adaptive memory in plants under diverse abiotic stresses.

In summary, DNA methylation serves as a universal yet highly plastic epigenetic interface through which plants perceive and adapt to a wide spectrum of abiotic stresses. The case studies from drought to heavy metal exposure demonstrate a common theme: stress signals are transduced into specific methylation patterns—often involving the RdDM pathway for establishment and CMTs for maintenance—that fine-tune the expression of key adaptive genes. Crucially, this reprogramming is not merely a transient response; it can be somatically memorized and, in many instances, transmitted across generations, thereby providing progeny with a heritable “priming” advantage. The collective evidence underscores that the DNA methylation system functions as a central decoder of environmental cues, enabling plants to implement precise, stress-tailored epigenetic strategies for survival and productivity (Figure 3).

This figure summarizes DNA methylation changes induced by eight abiotic stress types (drought, salinity, temperature, carbon, nutrient, light, ozone, and heavy metal stress), and their roles in establishing somatic and transgenerational memory.

## 4. Crop DNA Methylation Detection Technology Systems: Principles, Selection, and Integration Strategies

The accuracy and applicability of DNA methylation detection technologies are pivotal for translating epigenetic research into practical breeding applications. Based on throughput and target scope, existing techniques can be broadly classified into two main categories: targeted validation and panoramic scanning. The selection of an appropriate method hinges on balancing specific research objectives (e.g., candidate gene validation, epigenome mapping, or screening for population epigenetic variation) with the inherent characteristics of the technologies, including genomic coverage, base resolution, throughput, and cost [125].

### 4.1. Targeted Validation Technologies: High-Resolution Precision Guidance

These technologies are designed for analyzing the methylation status of specific genes or genomic loci, forming the foundation for functional validation studies. Their core principle predominantly relies on bisulfite conversion, a process that selectively deaminates unmethylated cytosines (C) to uracils (U, read as thymine, T, during sequencing) while leaving 5-methylcytosine (5mC) intact, thereby enabling the specific identification of methylated sites [126]. Bisulfite Sequencing PCR, based on this principle, is a commonly used method for validating dynamic methylation changes in key genes (e.g., stress-responsive genes) in crops like rice, owing to its technical maturity, relatively low cost, and reliability [127,128,129]. To circumvent potential issues associated with bisulfite treatment, such as DNA degradation, Methylation-Sensitive Restriction Enzyme analysis (e.g., MSRE-PCR) employs isoschizomers with differing sensitivity to methylation for DNA digestion, followed by PCR for rapid qualitative screening, making it suitable for the preliminary screening of large sample sets [130]. Furthermore, Methylation-Sensitive Amplification Polymorphism technology, based on the amplified fragment length polymorphism principle, allows semi-quantitative detection by exploiting differential enzyme sensitivity and was widely applied in early plant epigenetic research [131,132]. With the growing understanding of the functional roles of oxidized modifications like 5-hydroxymethylcytosine (5hmC), derivative techniques such as Oxidative Bisulfite Sequencing (oxBS-seq) and TET-Assisted Bisulfite Sequencing (TAB-seq) have been developed. These methods can specifically discriminate between 5mC and 5hmC, facilitating the study of more complex epigenetic dynamics [133,134]. Newer bisulfite-free techniques, including TET-Assisted Pyridine Borane Sequencing (TAPS) and Enzymatic Methyl Sequencing (EM-seq), replace chemical conversion with enzymatic reactions, minimizing DNA damage and improving both detection fidelity and the efficient use of DNA input [135,136]. The shared advantages of these targeted approaches are high resolution, strong specificity, and relatively straightforward data analysis. Their primary limitation lies in their restricted coverage, categorizing them as “hypothesis-driven” research tools that are less adept at discovering novel, unanticipated regulatory sites.

### 4.2. Panoramic Scanning Technologies: Genome-Wide Methylation Mapping

In contrast to targeted methods, panoramic scanning technologies aim to unbiasedly profile DNA methylation across the entire genome, which is crucial for discovering novel regulatory regions and constructing global epigenetic networks. Whole-Genome Bisulfite Sequencing (WGBS), capable of providing single-base resolution methylation information with near-complete genome coverage, is considered the “gold standard” for methylation analysis. It has been successfully employed to investigate tissue-specific methylation patterns in rice and epigenetic variations associated with domestication [137,138]. However, its high cost, complex bioinformatics analysis requirements, and the need for high DNA input pose limitations for its large-scale application in certain studies [139]. Reduced Representation Bisulfite Sequencing (RRBS) selectively enriches genomic regions rich in CpG dinucleotides and functionally relevant for gene regulation (e.g., CpG islands) using restriction enzymes (e.g., MspI), followed by bisulfite treatment and sequencing. This approach offers an efficient and cost-effective strategy for assessing methylation in key regulatory regions, making it suitable for population-level epigenome-wide association studies [140]. Another strategy is based on affinity enrichment. Techniques like Methylated DNA Immunoprecipitation Sequencing (MeDIP-seq) and its derivatives (e.g., Methyl-Capture Sequencing, MCS) utilize specific antibodies against 5mC or Methyl-CpG Binding Domain (MBD) proteins to capture methylated DNA fragments for subsequent sequencing [141,142]. The advantages of these methods include lower cost and reduced DNA input requirements. However, their spatial resolution is limited (typically in the range of 100–500 bp), rendering them more suitable for the rapid genome-wide identification and comparison of large differentially methylated regions [143].

### 4.3. Technology Integration Strategies and Future Perspectives

**Figure 4:**
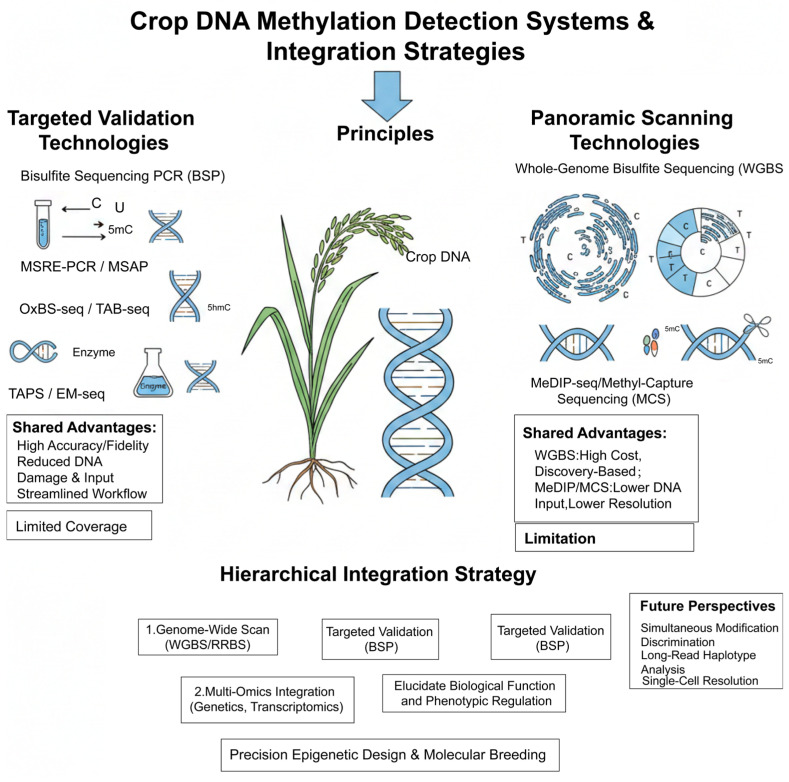
Framework for selection and integration of DNA methylation detection technologies in crop epigenetics and breeding.

Currently, no single technology perfectly balances the demands of high coverage, high resolution, and low cost. Consequently, a hierarchical integration strategy has become a mainstream paradigm in crop epigenetics, particularly for dissecting complex agronomic traits in species like rice. A typical workflow involves: first, conducting a genome-wide scan using WGBS or RRBS to identify trait-associated Differentially Methylated Regions (DMRs); second, employing targeted techniques like BSP for the fine validation and quantitative analysis of candidate DMRs; and, finally, integrating multi-omics data (e.g., genetics, transcriptomics) to systematically elucidate the biological function and phenotypic regulatory mechanisms of these methylation changes [144,145,146]. This “discovery–validation–mechanistic elucidation” closed-loop research model has significantly advanced our understanding of the epigenetic regulation underlying complex traits such as crop development and stress resistance. Future advancements in detection technology will likely focus on several key areas: first, developing sequencing methods capable of simultaneously and efficiently discriminating between various DNA modifications, including 5mC, 5hmC, and 5fC; second, leveraging third-generation long-read sequencing to enable methylation haplotype analysis at the single-molecule level, thereby addressing challenges in regions like repetitive sequences; third, advancing epigenomic techniques suitable for ultra-low input or even single-cell resolution to unravel precise epigenetic regulatory networks during processes involving tissue heterogeneity and dynamic development. These technological progresses will directly empower precision epigenetic design and molecular breeding in crops (Figure 4).

This figure presents the features of targeted validation technologies (BSP, MSAP, etc.) and panoramic scanning technologies (WGBS, RRBS, etc.), along with the “discovery–validation–mechanistic elucidation” closed-loop research strategy.

## 5. Applications of DNA Methylation in Plant Breeding

### 5.1. Breeding Resistant Cultivars Using Stress-Induced Heritable Epialleles

Environmental stresses can induce DNA methylation variations that are stably transmitted through meiosis, forming “epialleles,” which provide novel genetic resources for resistance breeding. Representative examples include, in rice, the hypomethylation state of the bacterial blight resistance gene *Xa21G* promoter, induced by either 5-azadeoxycytidine treatment or pathogen infection, and the conferred resistance can be stably inherited for at least nine generations [147]. Similarly, *Tobacco mosaic virus* (TMV) infection-induced genomic methylation changes and acquired resistance can also be transmitted to progeny [148]. These findings indicate that screening for or inducing favorable, heritable epialleles under stress conditions is an effective strategy for developing new disease-resistant and stress-tolerant varieties. Stress-induced epigenetic reprogramming often involves the RdDM pathway. This pathway can utilize stress-responsive small RNAs (sRNAs) to guide methyltransferases (e.g., *DRM2*) to specific genomic loci, thereby establishing or erasing DNA methylation marks [149,150]. Although many stress-induced epigenetic changes are dynamic, emerging synthetic apomixis technology offers a theoretical route to bypass meiosis and sexual recombination, enabling the direct cloning and fixation of F1 genotypes possessing desirable stress-resistant epigenetic states [151,152].

### 5.2. Targeted Gene Silencing Technologies Based on the RdDM Pathway and Methylation “Writers”

RdDM is a plant-specific pathway that utilizes small RNAs to guide de novo methylation at specific sequences [153]. The core enzymatic action of this pathway is carried out by methyltransferases like DOMAINS REARRANGED METHYLTRANSFERASE 2 (*DRM2*), which function as the “writers” of DNA methylation [154,155]. Leveraging this principle, virus-induced gene silencing (VIGS) technology has been adapted to direct the RdDM machinery to endogenous gene promoters, achieving heritable transcriptional silencing [156,157]. This provides a clear technical pathway for deliberately silencing genes detrimental to agronomic traits (e.g., genes delaying fruit ripening) through designed artificial sRNAs or viral vectors. The mechanistic details of the RdDM pathway are well elucidated, providing a foundation for more precise targeting. The core steps involve: the transcription of precursor RNA by Pol IV assisted by proteins like *SHH1* and *CLSY*; processing by *RDR2* and *DCL3* into 24-nt siRNAs; the loading of siRNAs into *AGO4/6/9* protein complexes for guiding to target sites; and the final recruitment of Pol V and the methyltransferase *DRM2* to establish de novo methylation at the target locus [158,159]. This process is tightly coupled with chromatin remodeling; for instance, *SUVH2/9* proteins recognize histone marks and cooperate with the DDR complex to recruit Pol V. These molecular insights pave the way for developing next-generation targeted silencing tools. Engineering key components of the RdDM pathway (e.g., *DRM2*, *AGO* proteins, or Pol V) holds promise for creating gene silencing systems with higher specificity and efficiency than conventional VIGS. Furthermore, given that RdDM often functions in concert with other epigenetic modifications like histone marks [160], future targeting strategies could consider simultaneous manipulation of both DNA methylation and histone modification states. This approach could achieve more stable and profound gene silencing, enabling the precise regulation of complex traits such as crop maturity, plant architecture, or secondary metabolite synthesis.

### 5.3. Harnessing DNA Methylation Polymorphisms to Decipher and Utilize Heterosis

Extensive DNA methylation polymorphisms exist between hybrid parents and in their hybrid offspring, and these epigenetic differences are likely implicated in heterosis (hybrid vigor). For example, methylation-sensitive amplification polymorphism (MSAP) analysis in rice hybrids and their parents revealed that hybrids exhibit significantly different methylation patterns at specific loci compared to their parents. In maize intra-species hybrids, approximately 6.59% to 11.92% of methylation sites display non-parental patterns [161]. Recent methylation analysis of soybean hybrid seeds (F0 generation) identified a large number of non-additive differentially methylated sites enriched in gene regions associated with transcriptional regulation and hormonal functions, suggesting their potential role in the early establishment of heterosis [162]. These findings indicate that whole-genome methylation profiles have potential as molecular markers for parental selection and heterosis prediction.

In-depth studies suggest that DNA methylation polymorphisms influence hybrid performance primarily through two mechanisms. First, hybridization-induced DNA methylation reprogramming can directly regulate the expression of a suite of genes associated with heterosis. In *Arabidopsis* and maize, methylation changes at specific sites (particularly in CHG and CHH contexts) post-hybridization are significantly correlated with trait advantages such as biomass and flowering time [163,164]. The affected genes are involved in key pathways including hormone signaling (e.g., *IAA*, *SAUR*), circadian rhythms (e.g., *CCA1*, *OsGI*), photosynthesis, and stress responses (e.g., H*SP70*, *WRKY*) [165]. Second, DNA methylation helps maintain post-hybridization genomic stability by effectively silencing transposable elements (TEs). Suppressing harmful TE activation is considered a conserved mechanism contributing to heterosis [166,167]. Therefore, integrating methylation profile analysis with multi-omics data (e.g., transcriptomics, epigenomics) enables a more systematic dissection of the complex regulatory network underlying heterosis.

**Figure 5:**
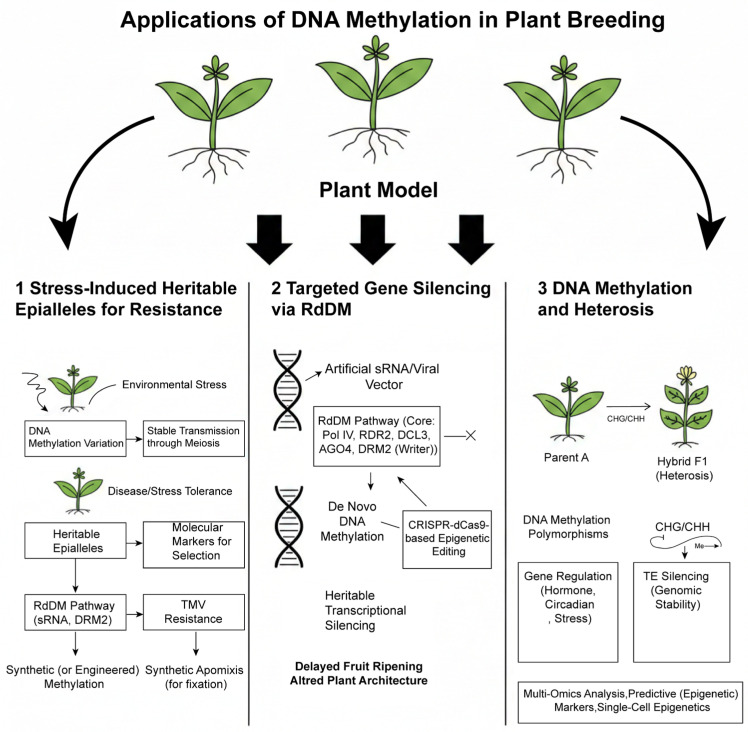
From epigenetic mechanisms to breeding applications: utilizing heritable epialleles, targeted silencing, and heterosis.

Looking forward, utilizing single-cell omics technologies and CRISPR-dCas9-based epigenetic editing tools [168], will allow researchers to precisely manipulate the methylation state of candidate loci in specific cell types or developmental stages. This will directly test the role of specific methylation variations in heterosis and ultimately guide precision parental selection and cross-design based on epigenetic markers (Figure 5).

This figure outlines three major pathways for translating DNA methylation research into breeding practices: the utilization of heritable epialleles, RdDM-based targeted gene silencing, and methylation marker-assisted heterosis prediction.

## 6. From Mechanisms to Applications: Translating DNA Methylation Research into Breeding Strategies

As the value of epigenetics in crop breeding becomes increasingly apparent, the central challenge lies in transforming frontier discoveries into predictable and operable breeding tools. Research on DNA methylation has advanced our understanding of how plants adapt to environmental stresses, yet its applied potential remains largely untapped. At present, this translational process faces critical hurdles in three interconnected areas: mechanistic understanding, technological capacity, and breeding integration.

### 6.1. From Correlation to Causation: Toward a Deeper Mechanistic Understanding

Existing studies have established broad correlations between DNA methylation patterns and stress phenotypes. For instance, mCG methylation near transcription start sites shows the strongest association with gene silencing, whereas the relationship between promoter methylation and gene expression is more complex, often defying simple repression–activation models [169,170]. However, among the numerous differentially methylated regions (DMRs) identified to date, distinguishing functionally relevant sites that actively contribute to stress adaptation from those that merely accompany stress responses remains a major challenge. Moving beyond correlative descriptions toward causal mechanistic insights will require new experimental paradigms. Single-cell epigenomics, for example, offers the possibility to trace the initiation and propagation of methylation dynamics across cell lineages and developmental timelines, thereby establishing temporal and causal links between epigenetic changes, downstream gene expression, and ultimately, stress phenotypes.

How environmental signals are “translated” into specific methylation patterns also remains an open question. Do different stresses—such as drought versus salinity—activate distinct upstream signaling pathways that differentially recruit or modulate components of the RdDM pathway (e.g., Pol IV/V, DRM2) or demethylases (e.g., ROS1)? Resolving these signaling nodes will be key to understanding the regulatory origins of epigenetic plasticity. For heritable epialleles that have been documented—such as those engineered at the OsFIE1 or ACT1 loci [171]—the molecular basis of their stability across generations is still poorly understood. This involves dissecting how epigenetic marks are erased, maintained, and re-established during meiosis. Only by clarifying these fundamental mechanisms can we begin to assess the long-term reliability of epigenetically modified traits in breeding programs.

### 6.2. Bridging the Technological Divide: Developing and Integrating Precision Tools

Technological limitations currently constrain both basic research and breeding applications. On the detection side, existing methods struggle to strike an ideal balance between genomic coverage, single-base resolution, and cost [172,173]. While emerging technologies such as long-read sequencing offer the potential to simultaneously capture multiple modifications—including 5mC, 5hmC, and others—along with their haplotype distribution, achieving functional interpretation of these marks within their three-dimensional chromatin context will require continued methodological innovation.

On the manipulation side, CRISPR-dCas9-based epigenome editing has proven feasible, but its translation into a robust breeding tool faces multiple hurdles related to efficiency, specificity, and persistence. Editing efficiencies in plants remain relatively low, and off-target effects—along with the stability of edited states during mitosis and meiosis—are difficult to predict with current tools [174,175,176]. A further layer of complexity arises from the fact that DNA methylation often functions in concert with other epigenetic marks such as histone modifications, and existing technologies cannot easily disentangle their respective contributions in vivo [177]. Developing next-generation editors that incorporate more specific effector domains, or exploring alternative delivery systems, will be essential for achieving precise reprogramming of defined chromatin states.

### 6.3. Building the Application Bridge: Toward an Epigenetic-Assisted Breeding Framework

Translating basic research and technological tools into breeding practice requires a clear integration pathway. A logical first step is the development of crop epigenomic resources and knowledge bases. This involves deep epigenome sequencing of core breeding germplasm to construct public resources akin to “epigenome reference panels” [178,179]. By combining genetic linkage analysis with genome-wide association studies, it is possible to systematically identify methylation quantitative trait loci (meQTLs)—genetic loci that control variation in specific methylation patterns [180]. Such efforts can anchor epigenetic variation to genomic coordinates and pinpoint stable, heritable epigenetic markers that may contribute to agronomic traits. For instance, population-wide methylome analysis in cotton has identified millions of cis-meQTLs and numerous trait-associated epigenetic loci independent of genetic variation, demonstrating the feasibility of integrating such markers into breeding strategies [180].

Building on this foundation, multi-layered breeding interventions become conceivable. For epigenetic targets with well-defined mechanisms and pronounced phenotypic effects—such as gene loci known to be involved in cold acclimation—editing tools can be deployed for directed optimization. For complex polygenic traits, identified epigenetic markers can be used for early prediction and selection. In hybrid breeding, parent-of-origin methylation differences might serve as novel predictors of heterosis. Looking further ahead, integrating genomic, epigenomic, and transcriptomic data into systems biology models could eventually enable a network-level understanding of complex traits such as broad adaptability, paving the way for more intelligent multi-environment selection strategies.

Taken together, the systematic elucidation of the complete pathway—from epigenetic signal perception to methylation remodeling, gene network regulation, physiological adaptation, and transgenerational inheritance—provides a theoretical foundation for molecular design breeding and signals a broader transition in DNA methylation research from a descriptive discipline toward a predictive engineering science. Although significant challenges remain, including gaps in mechanistic understanding and limitations in technological precision, the applied potential of epigenetics is gradually coming into view. By integrating epigenetic marker-assisted selection with epigenome editing and other advanced tools, it may become possible to develop next-generation stress-resilient crop varieties tailored to future climate scenarios, offering new solutions for global food security [181,182,183]. 

## Data Availability

No new data were created or analyzed in this study. Data sharing is not applicable to this article.
